# Crystal structure and Hirshfeld surface analysis of 1-(2,4-di­chloro­benz­yl)-5-methyl-*N*-(thio­phene-2-sulfon­yl)-1*H*-pyrazole-3-carboxamide

**DOI:** 10.1107/S2056989018006242

**Published:** 2018-04-27

**Authors:** Abdullah Aydin, Mehmet Akkurt, Zehra Tugce Gur, Erden Banoğlu

**Affiliations:** a Department of Mathematics and Science Education, Faculty of Education, Kastamonu University, 37200 Kastamonu, Turkey; b Department of Physics, Faculty of Sciences, Erciyes University, 38039 Kayseri, Turkey; c Department of Pharmaceutical Chemistry, Faculty of Pharmacy, Gazi University, 06330 Ankara, Turkey

**Keywords:** crystal structure, dimer, 1*H*-pyrazole ring, thio­phene ring, disorder, hydrogen-bonding patterns

## Abstract

In the title compound, C_16_H_13_Cl_2_N_3_O_3_S_2_, the thio­phene ring is disordered in a 0.762 (3):0.238 (3) ratio by an approximate 180° rotation of the ring around the S—C bond linking the ring to the sulfonyl unit. The di­chloro­benzene group is also disordered over two sets of sites with the same occupancy ratio. The mol­ecular conformation is stabilized by intra­molecular C—H⋯Cl and C—H⋯N hydrogen bonds, forming rings with graph-set notation *S*(5). In the crystal, pairs of mol­ecules are linked by N—H⋯O and C—H⋯O hydrogen bonds, forming inversion dimers with graph-set notation *R*_2_^2^(8) and *R*_1_^2^(11), which are connected by C—H⋯O hydrogen-bonding inter­actions into ribbons parallel to (100). The ribbons are further connected into a three-dimensional network by C—H⋯π inter­actions and π–π stacking inter­actions between benzene and thio­phene rings, with centroid-to-centroid distances of 3.865 (2), 3.867 (7) and 3.853 (2) Å. Hirshfeld surface analysis has been used to confirm and qu­antify the supra­molecular inter­actions.

## Abstract 2

In the crystal, pairs of mol­ecules are linked by N—H⋯O and C—H⋯O hydrogen bonds, forming inversion dimers with graph-set notation 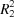
(8) and 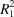
(11), which are connected by C—H⋯O hydrogen-bonding inter­actions into ribbons parallel to (100). The ribbons are further connected into a three-dimensional network by C—H⋯π inter­actions and π–π stacking inter­actions between benzene and the thio­phene rings.

## Chemical context   

The pyrazole *core* structure has been widely used as a common heterocyclic scaffold in medicinal chemistry to produce novel drug candidates with a great variety of pharmacological activities including anti-inflammatory, anti­platelet, anti­cancer, anti­mycobacterial, anti­depressant and anti­convulsant properties (Küçükgüzel & Şenkardeş, 2015 ▸; Çalışkan *et al.*, 2013 ▸; Ding *et al.*, 2009 ▸; Baraldi *et al.*, 2004 ▸; Palaska *et al.*, 2008 ▸). Among them, pyrazole-carboxamide derivatives have been shown to exhibit anti­mycobacterial, anti­fungal and anti­viral activities (Sun & Zhou, 2015 ▸; Yan *et al.*, 2018 ▸; Comber *et al.*, 1992 ▸). In the course of our ongoing research into bioactive pyrazole derivatives (Banoğlu *et al.*, 2005 ▸; Şüküroğlu *et al.*, 2005 ▸; Ergün *et al.*, 2010 ▸; Çalışkan *et al.*, 2011 ▸; Levent *et al.*, 2013 ▸; Cankara Pirol *et al.*, 2014 ▸), we have relied on the aforementioned biological properties of pyrazole-carboxamides and designed novel pyrazole-3-carboxamide derivatives for their potential anti­microbial activity. In this work, we report the crystallographic characterization and Hirshfeld surface analysis of one of these compounds bearing the 2,4-di­chloro­benzyl substituent at one of the pyrazole nitro­gen atoms.
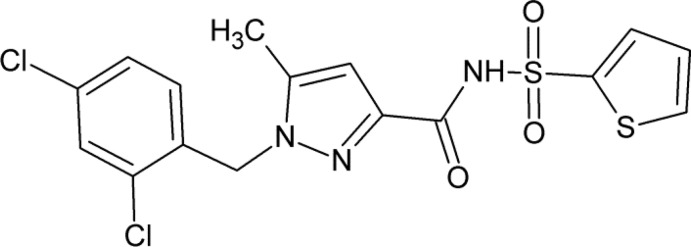


## Structural commentary   

**Figure 1:**
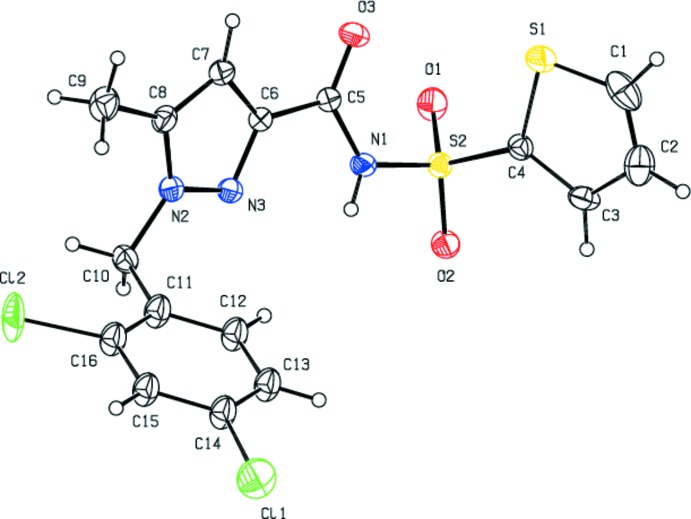
The mol­ecular structure of the title compound with displacement ellipsoids for non-H atoms drawn at the 30% probability level. The minor components of the disordered thio­phene and di­chloro­benzene groups have been omitted.

In the mol­ecule of the title compound (Fig. 1 ▸), the dihedral angles between the planes of the pyrazole ring *A* (N2/N3/C6–C8), the major and minor components *B* (S1/C1–C4) and *B*′ (S1*A*/C1/C2/C3*A*/C4) of the disordered thio­phene ring, and the disordered benzene ring C (C11–C16) and *C*′ (C11*A*–C16*A*) are *A*/*B* = 67.62 (16)°, *A*/*B*′ = 68.1 (5)°, *B*/*B*′ = 3.3 (5)°, *A*/*C* = 70.09 (16)°, *B*/*C* = 83.06 (19)° and *B*′/*C* = 80.2 (5)°, *A*/*C*′ = 78.4 (4)°, *B*/*C*′ = 77.3 (4)° and *B*′/*C*′ = 74.2 (6)°. The mol­ecular conformation is stabilized by intra­molecular C—H⋯Cl and C—H⋯N hydrogen bonds (Table 1 ▸), forming rings with graph-set notation *S*(5).

## Supra­molecular features   

**Figure 2:**
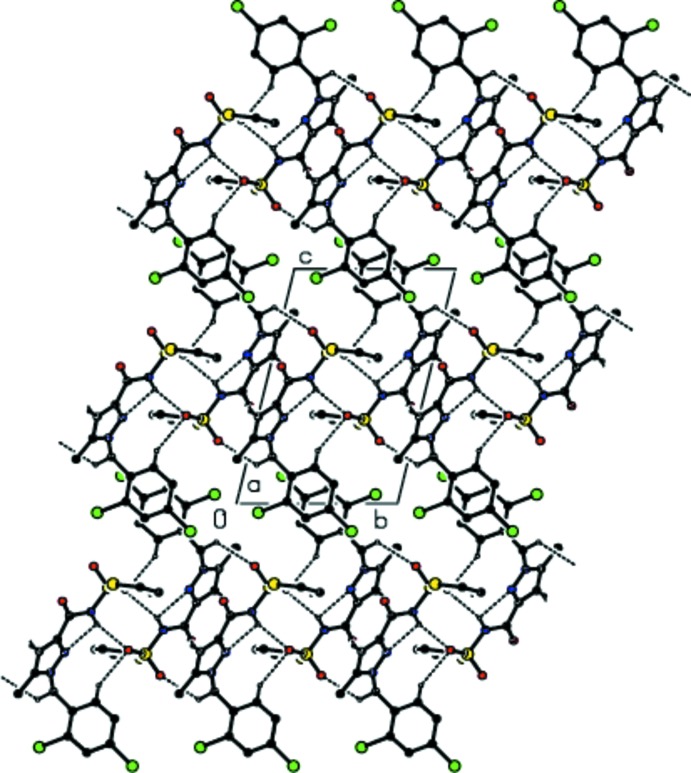
Crystal structure of the title compound viewed along the *a* axis. Dashed lines show hydrogen-bonding inter­actions. The minor components of the disordered groups have been omitted.

In the crystal, pairs of mol­ecules are linked by inter­molecular N—H⋯O and C—H⋯O hydrogen bonds (Table 1 ▸; Figs. 2 ▸ and 3 ▸), forming inversion dimers with graph-set notation 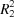
(8) and 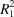
(11), which are connected by C—H⋯O hydrogen-bonding inter­actions into ribbons parallel to (100). The ribbons are further connected into a three-dimensional network by C—H⋯π inter­actions (Table 1 ▸) and π–π stacking inter­actions between the benzene and thio­phene rings, with centroid-to-centroid distances of 3.865 (2) Å for *Cg*1⋯*Cg*1^v^, 3.867 (7) Å for *Cg*2⋯*Cg*2^v^ and 3.853 (2) Å for *Cg*4⋯*Cg*4^vi^ where *Cg*1, *Cg*2 and *Cg*4 are the centroids of the thio­phene ring *B*, the thio­phene ring *B*′ and the benzene ring *C* [symmetry codes: (v) 2 − *x*, 1 − *y*, 1 − *z*; (vi) 1 − *x*, 1 − *y*, −*z*].

## Hirshfeld surface analysis   

**Figure 4:**
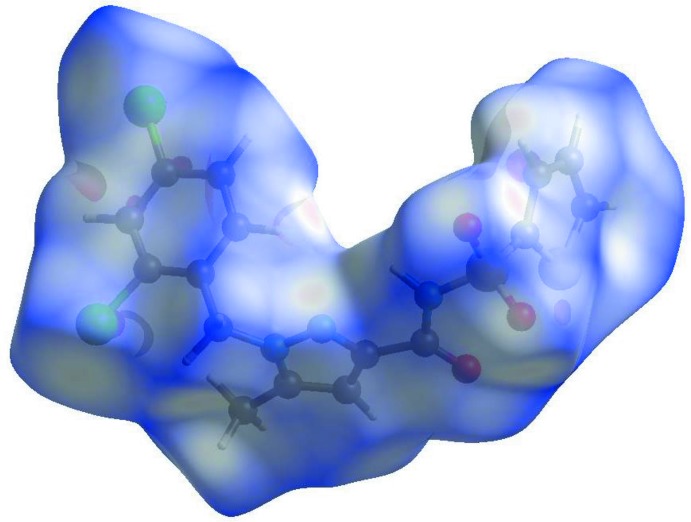
The Hirshfeld surface of the title compound mapped over *d*_norm_.

A Hirshfeld surface analysis (Hirshfeld, 1977 ▸; Spackman & Jayatilaka, 2009 ▸) of the title compound was carried out to investigate the location of atoms with potential to form hydrogen bonds and the qu­anti­tative ratio of these inter­actions. *CrystalExplorer17.5* (Turner *et al.*, 2017 ▸) was used to generate the Hirshfeld surface and two-dimensional fingerprint plots (Parkin *et al.*, 2007 ▸; Rohl *et al.*, 2008 ▸), using the atomic coordinates of the major disorder component of the disordered atoms (Figs. 4 ▸ and 5 ▸). The electrostatic potentials were calculated using *TONTO* (Spackman *et al.*, 2009 ▸) integrated into *CrystalExplorer*, wherein the respective experimental structure was used as the input to *TONTO*. Further, the electrostatic potentials were mapped on Hirshfeld surfaces using the STO-3G basis set at the Hartree–Fock level of theory.

The inter­molecular distance information on the surface can be condensed into a two-dimensional histogram of *d*_e_ and *d*_i_, which is a unique identifier for mol­ecules in a crystal structure, and is known as a fingerprint plot. Instead of plotting *d*_e_ and *d*_i_ on the Hirshfeld surface, contact distances are normalized in *CrystalExplorer* using the van der Waals radius of the appropriate inter­nal (*r*_i_^vdw^) and external (*r*_e_^vdw^) atom of the surface:

*d*_norm_= (*d*_i_ − *r*_i_^vdw^)/*r*_i_^vdw^ + (*d*_e_ − *r*_e_^vdw^)/*r_e_* ^vdw^.

**Figure 5:**
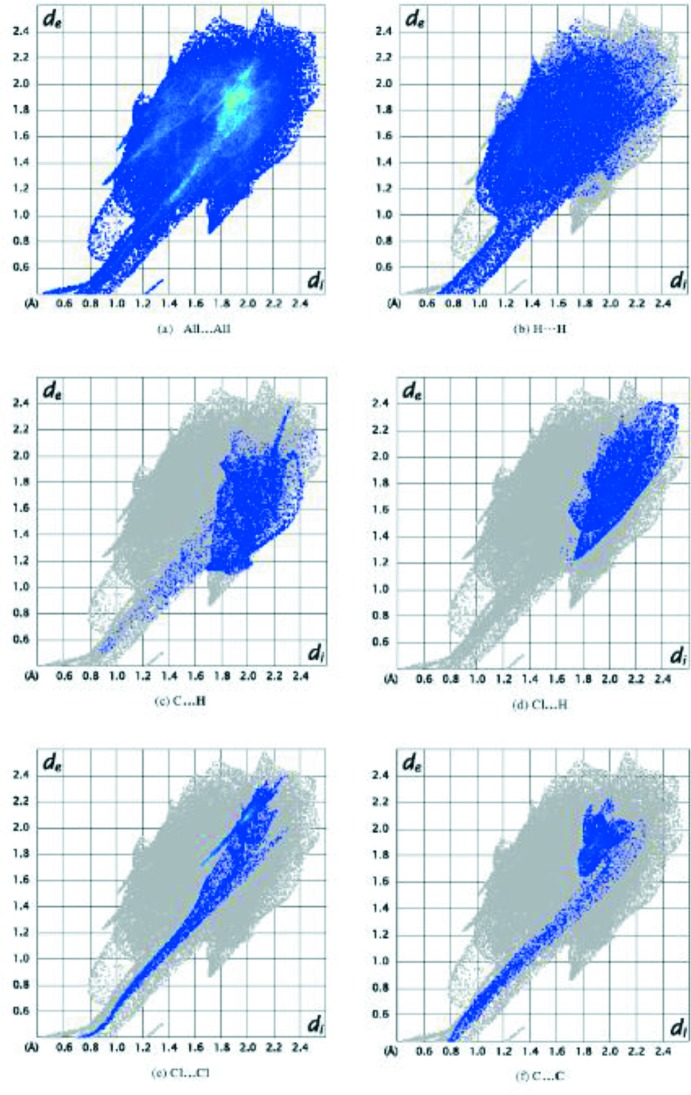
The two-dimensional fingerprint plots of the title compound, showing (*a*) all inter­actions, and delineated into (*b*) H⋯H, (*c*) C⋯H, (*d*) Cl⋯H, (*e*) Cl⋯Cl and (*f*) C⋯C inter­actions. The outline of the full fingerprint plots is shown in grey. *d*_i_ is the closest inter­nal distance from a given point on the Hirshfeld surface and *d*_e_ is the closest external contact.

The mol­ecular Hirshfeld surfaces were obtained using a standard (high) surface resolution with the three-dimentional *d*_norm_ surfaces mapped over a fixed colour scale of −1.9033 (red) to 1.1934 (blue). In the fingerprint plots (Rohl *et al.*, 2008 ▸), shown in Fig. 5 ▸, the points indicated by *b*, *c*, *d* and *e* correspond to H⋯H, C⋯H, Cl⋯H, Cl⋯Cl and C⋯C inter­actions with relative contributions of 28.4, 7.0, 6.8, 6.5 and 5.7%, respectively. These types of inter­actions add up to 54.4% of the inter­molecular contacts of the Hirshfeld surface area. The remaining contributions (8.3%) correspond to C⋯Cl (1.3%), N⋯C (1.3%) and other less important inter­actions (<1%). C⋯C contacts correspond to inter­molecular π–π inter­actions. The occurrence of non-high inter­action rates can be attributed to the fact that the small disordered portion of the mol­ecule is not considered.

## Database survey   

All bond lengths and angles are within normal ranges and are similar to those reported for related mol­ecules such as *trans*-*rac*-[1-oxo-2-phenethyl-3-(2-thien­yl)-1,2,3,4-tetra­hydro­iso­quin­olin-4-yl]methyl 4-methyl­benzene­sulfonate (Akkurt *et al.*, 2008 ▸), 2-benzene­sulfonamido­benzoic acid (Asiri *et al.*, 2009 ▸), propyl 2-(4-methyl­benzene­sulfonamido)­benzoate (Mustafa, Khan *et al.*, 2012 ▸), 2-{4-[acet­yl(eth­yl)amino]­benzene­sulfon­am­ido}­benzoic acid (Mustafa, Muhmood *et al.*, 2012 ▸), 2-(5-bromo­pyridin-3-yl)-5-[3-(4,5,6,7-tetra­hydro­thieno[3,2-*c*]pyridine-5-ylsulfon­yl)thio­phen-2-yl]-1,3,4-oxa­diazole (Fun *et al.*, 2011*a* ▸) and 2-(biphenyl-4-yl)-5-[3-(4,5,6,7-tetra­hydro­thieno[3,2-*c*]pyridine-5-ylsulfon­yl) thio­phen-2-yl]-1,3,4-oxa­diazole (Fun *et al.*, 2011*b* ▸).

## Synthesis and crystallization   

To a solution of methyl 1-(2,4-di­chloro­benz­yl)-5-methyl-1*H*-pyrazole-3-carboxyl­ate (200 mg, 0.70 mmol, 1 equiv.) in di­chloro­methane (DCM) were added 2-thio­phene­sulfonamide (126 mg, 0. 77 mmol, 1.1 equiv.), 1-ethyl-3-(3-di­methyl­amino-prop­yl)carbodi­imide (EDC; 148 mg, 0.77 mmol, 1.1 equiv.) and 4-dimethyl-amino­pyridine (DMAP; 17.8 mg, 0.14 mmol, 0.2 equiv.), and the resulting mixture was stirred overnight at room temperature. Upon completion of the reaction, the reaction mixture was partitioned between DCM and water. The collected organic layer was dried over anhydrous Na_2_SO_4_, filtered and evaporated to give the crude compound, which was purified with automated-flash chromatography (120.6 mg, 39.95%). The obtained product was recrystallized from hexane and ethyl acetate (4:1), m.p. 464.8–465.3 K. ^1^H NMR (CDCl_3_): δ 2.24 (3H, *s*), 5.33 (2H, *s*), 6.64 (2H, *m*), 7.12 (1H, *m*), 7.21 (1H, *dd*, *J* = 8.4, 2.1 Hz), 7.45 (1H, *d*, *J* = 2.1 Hz), 7.69 (1H, *dd*, *J* = 5.1, 1.2 Hz), 7.97 (1H, *dd*, *J* = 3.9, 1.2 Hz), 9.29 (1H, *bs*); ^13^C NMR (CDCl_3_): 11.2, 50.5, 107.6, 127.3, 127.9, 129.1, 129.6, 131.7, 132.9, 133.8, 134.8, 135.1, 139.2, 142.0, 143.5, 158.4. HRMS *m*/*z* calculated for C_16_H_13_Cl_2_N_3_O_3_S_2_ [*M* + H]^+^ 429.9854; found: 429.9857.

## Refinement details   

Crystal data, data collection and structure refinement details are summarized in Table 2 ▸. All H atoms bound to carbon atoms were positioned geometrically and treated as riding with C—H = 0.93-0.97 Å and *U*_iso_(H) = 1.2*U*_eq_(C) or 1.5*U*_eq_(C) for methyl H atoms. A rotating model was used for the methyl group. The nitro­gen-bound H atom (H1*N*) was located in a difference-Fourier map and refined with the constraint N1—H1*N* = 0.84 (3) Å and *U*_iso_(H) = 1.2*U*_eq_(N). The thio­phene ring is rotationally disordered by approximately 180° over two positions, the ratio of refined occupancies being 0.762 (3):0.238 (3). The di­chloro­benzene group of the title compound is also disordered over two sets of sites with the same occupancy ratio. The disordered dicholoro­benzene groups (*C*: C11–C16 and *C*′: C11*A*–C16*A*) were refined as rigid hexa­gons with bond lengths of 1.39 Å. The displacement ellipsoids for the corresponding carbon atoms in the disordered dicholoro­benzene groups were constrained by using the EADP command. Six outliers (633, 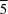
30, 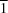
30, 515, 5
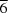
1, 520) were omitted in the final cycles of refinement.

## Supplementary Material

Crystal structure: contains datablock(s) global, I. DOI: 10.1107/S2056989018006242/rz5232sup1.cif

Structure factors: contains datablock(s) I. DOI: 10.1107/S2056989018006242/rz5232Isup3.hkl

Supporting information file. DOI: 10.1107/S2056989018006242/rz5232Isup4.cml

Checkcif Report. DOI: 10.1107/S2056989018006242/rz5232sup4.pdf

CCDC reference: 1839201

Additional supporting information:  crystallographic information; 3D view; checkCIF report

## Figures and Tables

**Figure 3:**
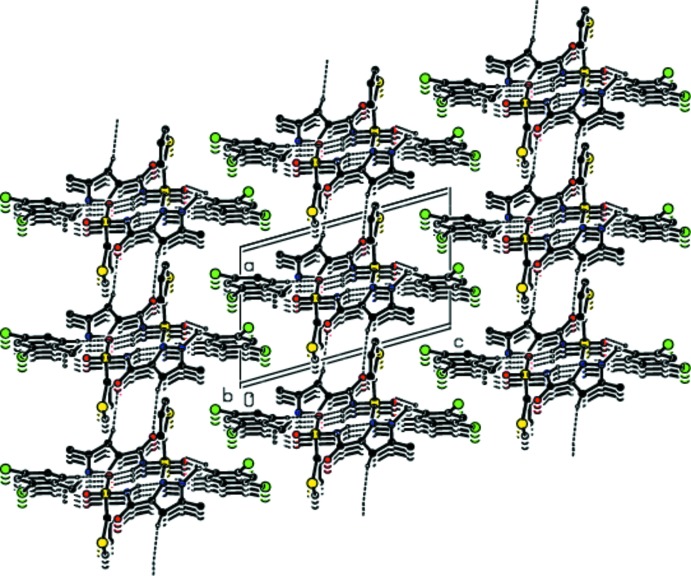
Crystal structure of the title compound viewed along the *b* axis. Dashed lines show hydrogen-bonding inter­actions. The minor components of the disordered groups have been omitted.

**Table 1:** Hydrogen-bond geometry (Å, °) *Cg*1 is the centroid of the major component (S1/C1–C4) of the disordered thio­phene ring.

*D*—H⋯*A*	*D*—H	H⋯*A*	*D*⋯*A*	*D*—H⋯*A*
N1—H1*N*⋯O2^i^	0.84 (3)	2.27 (3)	3.029 (3)	150 (3)
C7—H7⋯O3^ii^	0.93	2.59	3.437 (3)	152
C10—H10*B*⋯O1^iii^	0.97	2.52	3.141 (3)	122
C12—H12⋯N3	0.93	2.61	3.224 (3)	124
C12—H12⋯O2^i^	0.93	2.51	3.348 (3)	150
C15—H15⋯*Cg*1^iv^	0.93	2.97	3.893 (3)	174
C15*A*—H15*A*⋯*Cg*1^iv^	0.93	2.95	3.836 (8)	159

Symmetry codes: (i) 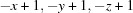; (ii) 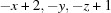; (iii) 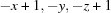; (iv) 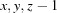.

**Table 2:** Experimental details

Crystal data	
Chemical formula	C_16_H_13_Cl_2_N_3_O_3_S_2_
*M*_r_	430.31
Crystal system, space group	Triclinic, *P* 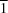
Temperature (K)	296
*a*, *b*, *c* (Å)	8.2706 (4), 8.7726 (4), 13.6433 (7)
α, β, γ (°)	76.091 (2), 74.610 (2), 87.970 (2)
*V* (Å^3^)	925.98 (8)
*Z*	2
Radiation type	Mo *K*α
μ (mm^−1^)	0.60
Crystal size (mm)	0.99 × 0.68 × 0.52

Data collection	
Diffractometer	Bruker APEXII CCD
Absorption correction	Multi-scan (*SADABS*; Bruker, 2007 ▸)
*T*_min_, *T*_max_	0.60, 0.75
No. of measured, independent and observed [*I* > 2σ(*I*)] reflections	19595, 4598, 4134
*R*_int_	0.024
(sin θ/λ)_max_ (Å^−1^)	0.668

Refinement	
*R*[*F* ^2^ > 2σ(*F* ^2^)], *wR*(*F* ^2^), *S*	0.056, 0.155, 1.03
No. of reflections	4598
No. of parameters	216
No. of restraints	14
H-atom treatment	H atoms treated by a mixture of independent and constrained refinement
Δρ_max_, Δρ_min_ (e Å^−3^)	1.20, −0.82

Computer programs: *APEX2* and *SAINT* (Bruker, 2007 ▸), *SHELXT2014* (Sheldrick, 2015*a* ▸), *SHELXL2014* (Sheldrick, 2015*b* ▸), *ORTEP-3 for Windows* and *WinGX* (Farrugia, 2012 ▸) and *PLATON* (Spek, 2009 ▸).

## supplementary crystallographic information

### Crystal data

**Table:**

C_16_H_13_Cl_2_N_3_O_3_S_2_	*Z* = 2
*M**_r_* = 430.31	*F*(000) = 440
Triclinic, *P*1	*D*_x_ = 1.543 Mg m^−^^3^
Hall symbol: -P 1	Mo *K*α radiation, λ = 0.71073 Å
*a* = 8.2706 (4) Å	Cell parameters from 9888 reflections
*b* = 8.7726 (4) Å	θ = 2.4–28.3°
*c* = 13.6433 (7) Å	µ = 0.60 mm^−^^1^
α = 76.091 (2)°	*T* = 296 K
β = 74.610 (2)°	Prism, translucent light white
γ = 87.970 (2)°	0.99 × 0.68 × 0.52 mm
*V* = 925.98 (8) Å^3^	

### Data collection

**Table:**

Bruker APEXII CCD diffractometer	4598 independent reflections
Radiation source: sealed tube	4134 reflections with *I* > 2σ(*I*)
Graphite monochromator	*R*_int_ = 0.024
φ and ω scans	θ_max_ = 28.4°, θ_min_ = 1.6°
Absorption correction: multi-scan (SADABS; Bruker, 2007)	*h* = −10→11
*T*_min_ = 0.60, *T*_max_ = 0.75	*k* = −11→11
19595 measured reflections	*l* = −17→18

### Refinement

**Table:**

Refinement on *F*^2^	14 restraints
Least-squares matrix: full	Hydrogen site location: mixed
*R*[*F*^2^ > 2σ(*F*^2^)] = 0.056	H atoms treated by a mixture of independent and constrained refinement
*wR*(*F*^2^) = 0.155	*w* = 1/[σ^2^(*F*_o_^2^) + (0.082*P*)^2^ + 0.8819*P*] where *P* = (*F*_o_^2^ + 2*F*_c_^2^)/3
*S* = 1.03	(Δ/σ)_max_ < 0.001
4598 reflections	Δρ_max_ = 1.20 e Å^−^^3^
216 parameters	Δρ_min_ = −0.82 e Å^−^^3^

### Special details

**Table:**

Geometry. Bond distances, angles etc. have been calculated using the rounded fractional coordinates. All su's are estimated from the variances of the (full) variance-covariance matrix. The cell esds are taken into account in the estimation of distances, angles and torsion angles
Refinement. Refinement on F^2^ for ALL reflections except those flagged by the user for potential systematic errors. Weighted R-factors wR and all goodnesses of fit S are based on F^2^, conventional R-factors R are based on F, with F set to zero for negative F^2^. The observed criterion of F^2^ > 2sigma(F^2^) is used only for calculating -R-factor-obs etc. and is not relevant to the choice of reflections for refinement. R-factors based on F^2^ are statistically about twice as large as those based on F, and R-factors based on ALL data will be even larger.

### Fractional atomic coordinates and isotropic or equivalent isotropic displacement parameters (Å^2^)

**Table:**

	*x*	*y*	*z*	*U*_iso_*/*U*_eq_	Occ. (<1)
C1	1.0018 (3)	0.5302 (5)	0.6433 (2)	0.0643 (8)	
H1	1.109594	0.540142	0.650187	0.077*	
C2	0.9085 (4)	0.6538 (4)	0.6217 (3)	0.0615 (8)	
H2	0.943899	0.757761	0.609474	0.074*	
C3	0.7555 (6)	0.6082 (6)	0.6199 (4)	0.0426 (11)	0.762 (3)
H3	0.670625	0.678061	0.610153	0.051*	0.762 (3)
S1	0.91332 (19)	0.35743 (15)	0.65722 (17)	0.0492 (3)	0.762 (3)
C3A	0.901 (2)	0.382 (2)	0.655 (2)	0.0426 (11)	0.238 (3)
H3A	0.933907	0.278893	0.670858	0.051*	0.238 (3)
S1A	0.7356 (7)	0.6272 (6)	0.6034 (4)	0.0492 (3)	0.238 (3)
C4	0.7359 (3)	0.4413 (3)	0.63434 (16)	0.0334 (4)	
C5	0.7050 (3)	0.1354 (2)	0.52207 (17)	0.0330 (4)	
C6	0.7152 (3)	0.0968 (2)	0.42142 (16)	0.0313 (4)	
C7	0.8307 (3)	0.0022 (3)	0.37032 (19)	0.0375 (5)	
H7	0.916655	−0.053427	0.393564	0.045*	
C8	0.7892 (3)	0.0094 (3)	0.27811 (18)	0.0361 (4)	
C9	0.8648 (4)	−0.0637 (4)	0.1890 (2)	0.0529 (6)	
H9A	0.794588	−0.150751	0.192409	0.079*	
H9B	0.974150	−0.100242	0.193074	0.079*	
H9C	0.874250	0.012686	0.124042	0.079*	
C10	0.5646 (3)	0.1512 (3)	0.19870 (18)	0.0397 (5)	
H10A	0.448790	0.169220	0.231841	0.048*	
H10B	0.565068	0.066145	0.164493	0.048*	
C11	0.6400 (5)	0.2998 (3)	0.1159 (2)	0.0466 (3)	0.762 (3)
C12	0.6577 (4)	0.4311 (3)	0.15290 (16)	0.0466 (3)	0.762 (3)
H12	0.629582	0.423813	0.224578	0.056*	0.762 (3)
C13	0.7173 (4)	0.5732 (3)	0.08277 (18)	0.0466 (3)	0.762 (3)
H13	0.729125	0.660962	0.107532	0.056*	0.762 (3)
C14	0.7593 (4)	0.5840 (2)	−0.02436 (17)	0.0466 (3)	0.762 (3)
C15	0.7416 (5)	0.4528 (3)	−0.06137 (17)	0.0466 (3)	0.762 (3)
H15	0.769664	0.460061	−0.133046	0.056*	0.762 (3)
C16	0.6819 (6)	0.3107 (3)	0.0088 (3)	0.0466 (3)	0.762 (3)
Cl1	0.8269 (3)	0.7564 (2)	−0.11887 (17)	0.0835 (5)	0.762 (3)
Cl2	0.6424 (5)	0.1501 (4)	−0.0388 (2)	0.0805 (7)	0.762 (3)
C11A	0.6465 (15)	0.3022 (11)	0.1193 (7)	0.0466 (3)	0.238 (3)
C12A	0.7063 (12)	0.4320 (11)	0.1423 (5)	0.0466 (3)	0.238 (3)
H12A	0.696770	0.433593	0.211501	0.056*	0.238 (3)
C13A	0.7802 (12)	0.5595 (9)	0.0618 (6)	0.0466 (3)	0.238 (3)
H13A	0.820202	0.646324	0.077136	0.056*	0.238 (3)
C14A	0.7945 (12)	0.5571 (9)	−0.0417 (5)	0.0466 (3)	0.238 (3)
C15A	0.7347 (15)	0.4273 (11)	−0.0646 (6)	0.0466 (3)	0.238 (3)
H15A	0.744229	0.425767	−0.133838	0.056*	0.238 (3)
C16A	0.6608 (17)	0.2999 (11)	0.0159 (9)	0.0466 (3)	0.238 (3)
Cl1A	0.8896 (10)	0.7355 (9)	−0.1136 (7)	0.0835 (5)	0.238 (3)
Cl2A	0.6826 (18)	0.1514 (15)	−0.0300 (9)	0.0805 (7)	0.238 (3)
N1	0.6005 (2)	0.2605 (2)	0.53833 (15)	0.0363 (4)	
H1N	0.571 (4)	0.317 (3)	0.4869 (18)	0.044*	
N2	0.6550 (2)	0.1036 (2)	0.27928 (14)	0.0340 (4)	
N3	0.6080 (2)	0.1593 (2)	0.36579 (14)	0.0334 (4)	
O1	0.5273 (2)	0.2101 (2)	0.73271 (13)	0.0479 (4)	
O2	0.4310 (2)	0.4460 (2)	0.62514 (13)	0.0412 (4)	
O3	0.7814 (2)	0.0698 (2)	0.58384 (14)	0.0473 (4)	
S2	0.55880 (6)	0.33499 (6)	0.64141 (4)	0.03159 (15)	

### Atomic displacement parameters (Å^2^)

**Table:**

	*U*^11^	*U*^22^	*U*^33^	*U*^12^	*U*^13^	*U*^23^
Cl1	0.1094 (16)	0.0562 (7)	0.0754 (7)	−0.0290 (10)	−0.0317 (10)	0.0150 (5)
Cl1A	0.1094 (16)	0.0562 (7)	0.0754 (7)	−0.0290 (10)	−0.0317 (10)	0.0150 (5)
Cl2	0.144 (2)	0.0649 (5)	0.0438 (7)	−0.0339 (10)	−0.0276 (8)	−0.0255 (5)
Cl2A	0.144 (2)	0.0649 (5)	0.0438 (7)	−0.0339 (10)	−0.0276 (8)	−0.0255 (5)
S1	0.0461 (5)	0.0507 (7)	0.0605 (6)	0.0166 (4)	−0.0260 (4)	−0.0205 (6)
S1A	0.0461 (5)	0.0507 (7)	0.0605 (6)	0.0166 (4)	−0.0260 (4)	−0.0205 (6)
S2	0.0325 (3)	0.0336 (3)	0.0302 (3)	0.0020 (2)	−0.0086 (2)	−0.0102 (2)
O1	0.0609 (11)	0.0441 (9)	0.0348 (8)	−0.0091 (8)	−0.0098 (7)	−0.0038 (7)
O2	0.0342 (8)	0.0474 (9)	0.0468 (9)	0.0105 (7)	−0.0122 (7)	−0.0204 (7)
O3	0.0556 (10)	0.0487 (10)	0.0485 (10)	0.0187 (8)	−0.0304 (8)	−0.0160 (8)
N1	0.0458 (10)	0.0360 (9)	0.0336 (9)	0.0126 (8)	−0.0186 (8)	−0.0135 (7)
N2	0.0386 (9)	0.0332 (8)	0.0323 (9)	0.0031 (7)	−0.0116 (7)	−0.0099 (7)
N3	0.0366 (9)	0.0338 (8)	0.0330 (9)	0.0061 (7)	−0.0125 (7)	−0.0108 (7)
C1	0.0361 (12)	0.105 (3)	0.0567 (17)	−0.0026 (14)	−0.0139 (11)	−0.0267 (17)
C2	0.0577 (16)	0.0581 (17)	0.0667 (19)	−0.0200 (14)	−0.0091 (14)	−0.0164 (14)
C3	0.0345 (19)	0.0394 (19)	0.051 (2)	−0.0035 (14)	−0.0204 (15)	0.0060 (15)
C3A	0.0345 (19)	0.0394 (19)	0.051 (2)	−0.0035 (14)	−0.0204 (15)	0.0060 (15)
C4	0.0306 (9)	0.0381 (10)	0.0336 (10)	0.0034 (8)	−0.0096 (7)	−0.0122 (8)
C5	0.0344 (10)	0.0310 (9)	0.0369 (10)	0.0036 (7)	−0.0137 (8)	−0.0103 (8)
C6	0.0332 (9)	0.0285 (9)	0.0344 (10)	0.0027 (7)	−0.0119 (8)	−0.0088 (8)
C7	0.0361 (10)	0.0351 (10)	0.0468 (12)	0.0078 (8)	−0.0157 (9)	−0.0158 (9)
C8	0.0372 (10)	0.0317 (10)	0.0411 (11)	0.0031 (8)	−0.0091 (8)	−0.0139 (8)
C9	0.0538 (14)	0.0576 (15)	0.0525 (15)	0.0090 (12)	−0.0087 (12)	−0.0298 (13)
C10	0.0471 (12)	0.0413 (11)	0.0348 (11)	−0.0009 (9)	−0.0173 (9)	−0.0094 (9)
C11	0.0639 (8)	0.0396 (6)	0.0367 (6)	−0.0054 (6)	−0.0098 (5)	−0.0130 (5)
C11A	0.0639 (8)	0.0396 (6)	0.0367 (6)	−0.0054 (6)	−0.0098 (5)	−0.0130 (5)
C12	0.0639 (8)	0.0396 (6)	0.0367 (6)	−0.0054 (6)	−0.0098 (5)	−0.0130 (5)
C12A	0.0639 (8)	0.0396 (6)	0.0367 (6)	−0.0054 (6)	−0.0098 (5)	−0.0130 (5)
C13	0.0639 (8)	0.0396 (6)	0.0367 (6)	−0.0054 (6)	−0.0098 (5)	−0.0130 (5)
C13A	0.0639 (8)	0.0396 (6)	0.0367 (6)	−0.0054 (6)	−0.0098 (5)	−0.0130 (5)
C14	0.0639 (8)	0.0396 (6)	0.0367 (6)	−0.0054 (6)	−0.0098 (5)	−0.0130 (5)
C14A	0.0639 (8)	0.0396 (6)	0.0367 (6)	−0.0054 (6)	−0.0098 (5)	−0.0130 (5)
C15	0.0639 (8)	0.0396 (6)	0.0367 (6)	−0.0054 (6)	−0.0098 (5)	−0.0130 (5)
C15A	0.0639 (8)	0.0396 (6)	0.0367 (6)	−0.0054 (6)	−0.0098 (5)	−0.0130 (5)
C16	0.0639 (8)	0.0396 (6)	0.0367 (6)	−0.0054 (6)	−0.0098 (5)	−0.0130 (5)
C16A	0.0639 (8)	0.0396 (6)	0.0367 (6)	−0.0054 (6)	−0.0098 (5)	−0.0130 (5)

### Geometric parameters (Å, º)

**Table:**

Cl1—C14	1.733 (3)	C10—C11	1.530 (4)
Cl1A—C14A	1.723 (12)	C11—C12	1.391 (4)
Cl2—C16	1.760 (5)	C11—C16	1.390 (5)
Cl2A—C16A	1.561 (17)	C11A—C16A	1.389 (15)
S1—C4	1.688 (3)	C11A—C12A	1.391 (14)
S1—C1	1.654 (4)	C12—C13	1.390 (4)
S1A—C4	1.583 (6)	C12A—C13A	1.390 (11)
S1A—C2	1.550 (7)	C13—C14	1.390 (3)
S2—O2	1.4362 (18)	C13A—C14A	1.391 (10)
S2—N1	1.641 (2)	C14—C15	1.390 (3)
S2—C4	1.733 (3)	C14A—C15A	1.390 (13)
S2—O1	1.4184 (18)	C15—C16	1.390 (4)
O3—C5	1.210 (3)	C15A—C16A	1.390 (14)
N1—C5	1.396 (3)	C1—H1	0.9300
N2—N3	1.343 (3)	C2—H2	0.9300
N2—C8	1.359 (3)	C3—H3	0.9300
N2—C10	1.461 (3)	C3A—H3A	0.9300
N3—C6	1.336 (3)	C7—H7	0.9300
C1—C2	1.329 (5)	C9—H9A	0.9600
C1—C3A	1.522 (19)	C9—H9C	0.9600
N1—H1N	0.84 (3)	C9—H9B	0.9600
C2—C3	1.349 (6)	C10—H10A	0.9700
C3—C4	1.439 (6)	C10—H10B	0.9700
C3A—C4	1.516 (19)	C12—H12	0.9300
C5—C6	1.472 (3)	C12A—H12A	0.9300
C6—C7	1.399 (3)	C13—H13	0.9300
C7—C8	1.376 (3)	C13A—H13A	0.9300
C8—C9	1.491 (4)	C15—H15	0.9300
C10—C11A	1.540 (10)	C15A—H15A	0.9300
			
C1—S1—C4	91.91 (16)	Cl1—C14—C15	115.95 (18)
C2—S1A—C4	96.3 (4)	C13—C14—C15	120.0 (2)
O1—S2—N1	108.73 (10)	Cl1A—C14A—C13A	104.5 (7)
O1—S2—C4	108.83 (11)	Cl1A—C14A—C15A	135.5 (6)
O1—S2—O2	120.05 (10)	C13A—C14A—C15A	120.0 (7)
O2—S2—C4	107.17 (12)	C14—C15—C16	120.0 (2)
N1—S2—C4	107.33 (10)	C14A—C15A—C16A	120.0 (8)
O2—S2—N1	104.06 (10)	Cl2—C16—C11	120.2 (3)
S2—N1—C5	126.32 (16)	Cl2—C16—C15	119.6 (3)
N3—N2—C8	112.91 (18)	C11—C16—C15	120.0 (3)
N3—N2—C10	119.06 (18)	C11A—C16A—C15A	120.0 (10)
C8—N2—C10	128.01 (19)	Cl2A—C16A—C11A	126.3 (10)
N2—N3—C6	104.17 (16)	Cl2A—C16A—C15A	107.6 (9)
S1—C1—C2	115.5 (2)	S1—C1—H1	122.00
C2—C1—C3A	108.6 (7)	C2—C1—H1	122.00
S2—N1—H1N	113.9 (18)	C3A—C1—H1	129.00
C5—N1—H1N	118.5 (18)	S1A—C2—H2	116.00
C1—C2—C3	110.9 (4)	C1—C2—H2	125.00
S1A—C2—C1	118.7 (4)	C3—C2—H2	125.00
C2—C3—C4	113.7 (4)	C2—C3—H3	123.00
C1—C3A—C4	104.5 (12)	C4—C3—H3	123.00
S1—C4—C3	107.9 (3)	C4—C3A—H3A	128.00
S2—C4—C3A	129.1 (7)	C1—C3A—H3A	128.00
S1A—C4—S2	119.4 (3)	C8—C7—H7	128.00
S2—C4—C3	128.3 (3)	C6—C7—H7	128.00
S1—C4—S2	123.50 (17)	C8—C9—H9A	109.00
S1A—C4—C3A	111.6 (8)	H9A—C9—H9B	109.00
O3—C5—C6	124.5 (2)	C8—C9—H9B	110.00
O3—C5—N1	122.7 (2)	C8—C9—H9C	110.00
N1—C5—C6	112.78 (19)	H9B—C9—H9C	109.00
C5—C6—C7	128.2 (2)	H9A—C9—H9C	109.00
N3—C6—C7	111.90 (19)	N2—C10—H10B	109.00
N3—C6—C5	119.91 (19)	C11—C10—H10A	109.00
C6—C7—C8	104.9 (2)	C11A—C10—H10A	110.00
C7—C8—C9	131.6 (3)	C11A—C10—H10B	111.00
N2—C8—C9	122.3 (2)	C11—C10—H10B	109.00
N2—C8—C7	106.1 (2)	N2—C10—H10A	109.00
N2—C10—C11A	110.0 (5)	H10A—C10—H10B	108.00
N2—C10—C11	113.2 (2)	C13—C12—H12	120.00
C10—C11—C12	116.2 (2)	C11—C12—H12	120.00
C10—C11—C16	123.7 (3)	C13A—C12A—H12A	120.00
C12—C11—C16	120.0 (2)	C11A—C12A—H12A	120.00
C10—C11A—C12A	126.4 (7)	C14—C13—H13	120.00
C10—C11A—C16A	113.6 (8)	C12—C13—H13	120.00
C12A—C11A—C16A	120.0 (9)	C14A—C13A—H13A	120.00
C11—C12—C13	120.0 (2)	C12A—C13A—H13A	120.00
C11A—C12A—C13A	120.0 (7)	C16—C15—H15	120.00
C12—C13—C14	120.0 (2)	C14—C15—H15	120.00
C12A—C13A—C14A	120.0 (8)	C14A—C15A—H15A	120.00
Cl1—C14—C13	123.98 (18)	C16A—C15A—H15A	120.00
			
C1—S1—C4—S2	−176.25 (16)	C2—C3—C4—S1	3.8 (5)
C1—S1—C4—C3	−2.0 (3)	C2—C3—C4—S2	177.7 (3)
C4—S1—C1—C2	−0.1 (3)	O3—C5—C6—C7	−12.2 (4)
O1—S2—N1—C5	−44.1 (2)	N1—C5—C6—N3	−11.7 (3)
O2—S2—N1—C5	−173.15 (18)	N1—C5—C6—C7	166.0 (2)
C4—S2—N1—C5	73.5 (2)	O3—C5—C6—N3	170.1 (2)
O2—S2—C4—S1	175.63 (16)	N3—C6—C7—C8	0.1 (3)
N1—S2—C4—S1	−73.11 (18)	C5—C6—C7—C8	−177.8 (2)
O1—S2—C4—C3	−128.6 (3)	C6—C7—C8—C9	179.3 (3)
O2—S2—C4—C3	2.6 (3)	C6—C7—C8—N2	−0.5 (3)
N1—S2—C4—C3	113.9 (3)	N2—C10—C11—C12	−55.0 (4)
O1—S2—C4—S1	44.39 (19)	N2—C10—C11—C16	128.4 (4)
S2—N1—C5—C6	179.75 (16)	C10—C11—C12—C13	−176.8 (3)
S2—N1—C5—O3	−2.0 (3)	C16—C11—C12—C13	0.0 (6)
C8—N2—C10—C11	−88.0 (3)	C10—C11—C16—Cl2	2.2 (6)
C8—N2—N3—C6	−0.6 (2)	C10—C11—C16—C15	176.6 (4)
N3—N2—C8—C7	0.7 (3)	C12—C11—C16—Cl2	−174.4 (3)
N3—N2—C10—C11	90.2 (2)	C12—C11—C16—C15	0.0 (7)
C10—N2—C8—C7	178.9 (2)	C11—C12—C13—C14	0.0 (5)
C10—N2—N3—C6	−179.01 (19)	C12—C13—C14—Cl1	177.4 (3)
C10—N2—C8—C9	−0.8 (4)	C12—C13—C14—C15	0.0 (5)
N3—N2—C8—C9	−179.1 (2)	Cl1—C14—C15—C16	−177.6 (3)
N2—N3—C6—C5	178.40 (18)	C13—C14—C15—C16	0.0 (6)
N2—N3—C6—C7	0.3 (2)	C14—C15—C16—Cl2	174.4 (3)
S1—C1—C2—C3	2.4 (4)	C14—C15—C16—C11	0.0 (7)
C1—C2—C3—C4	−4.0 (5)		

### Hydrogen-bond geometry (Å, º)

Cg1 is the centroid of the major component (S1/C1–C4) of the disordered thiophene ring.

**Table:**

*D*—H···*A*	*D*—H	H···*A*	*D*···*A*	*D*—H···*A*
N1—H1*N*···N3	0.84 (3)	2.35 (3)	2.694 (3)	105 (2)
N1—H1*N*···O2^i^	0.84 (3)	2.27 (3)	3.029 (3)	150 (3)
C7—H7···O3^ii^	0.93	2.59	3.437 (3)	152
C10—H10*B*···Cl2	0.97	2.60	3.134 (4)	115
C10—H10*B*···Cl2*A*	0.97	2.50	3.012 (12)	112
C10—H10*B*···O1^iii^	0.97	2.52	3.141 (3)	122
C12—H12···N3	0.93	2.61	3.224 (3)	124
C12—H12···O2^i^	0.93	2.51	3.348 (3)	150
C15—H15···*Cg*1^iv^	0.93	2.97	3.893 (3)	174
C15*A*—H15*A*···*Cg*1^iv^	0.93	2.95	3.836 (8)	159

Symmetry codes: (i) −*x*+1, −*y*+1, −*z*+1; (ii) −*x*+2, −*y*, −*z*+1; (iii) −*x*+1, −*y*, −*z*+1; (iv) *x*, *y*, *z*−1.
