# Supplementary material for: Crystal structure and Hirshfeld surface analysis of 1-(2,4-di­chloro­benz­yl)-5-methyl-N-(thio­phene-2-sulfon­yl)-1H-pyrazole-3-carboxamide
Source: Acta Crystallogr E Crystallogr Commun. 2018 Apr 27;74(Pt 5):747–51. doi: 10.1107/S2056989018006242 (PMC5947501; doi:10.1107/S2056989018006242)

checkCIF/PLATON report

Structure factors have been supplied for datablock(s) I

THIS REPORT IS FOR GUIDANCE ONLY. IF USED AS PART OF A REVIEW PROCEDURE FOR PUBLICATION, IT SHOULD NOT REPLACE THE EXPERTISE OF AN EXPERIENCED CRYSTALLOGRAPHIC REFEREE.

No syntax errors found. CIF dictionary Interpreting this report

Datablock: I

```
Bond precision:  C-C = 0.0036 A          Wavelength=0.71073

Cell:          a=8.2706(4)       b=8.7726(4)      c=13.6433(7)
               alpha=76.091(2)   beta=74.610(2)   gamma=87.970(2)
Temperature:   296 K

                Calculated                 Reported
Volume          925.98(8)                  925.98(8)
Space group     P -1                       P -1
Hall group      -P 1                       -P 1
Moiety formula  C16 H13 Cl2 N3 O3 S2       C16 H13 Cl2 N3 O3 S2
Sum formula     C16 H13 Cl2 N3 O3 S2       C16 H13 Cl2 N3 O3 S2
Mr              430.31                     430.31
Dx,g cm-3       1.543                      1.543
Z               2                          2
Mu (mm-1)       0.598                      0.598
F000            440.0                      440.0
F000'           441.24
h,k,lmax        11,11,18                   11,11,18
Nref            4627                       4598
Tmin,Tmax       0.622,0.734                0.600,0.750
Tmin'           0.549

Correction method= # Reported T Limits: Tmin=0.600 Tmax=0.750
AbsCorr = MULTI-SCAN

Data completeness= 0.994          Theta(max)= 28.350

R(reflections)= 0.0561( 4134)     wR2(reflections)= 0.1554( 4598)

S = 1.030                 Npar= 216
```

The following ALERTS were generated. Each ALERT has the format
test-name_ALERT_alert-type_alert-level.
Click on the hyperlinks for more details of the test.

Alert level C

```
PLAT911_ALERT_3_C Missing FCF Refl Between Thmin & STh/L=    0.600          6 Report
PLAT918_ALERT_3_C Reflection(s) with I(obs) much Smaller I(calc) .          2 Check
```

Alert level G

```
PLAT002_ALERT_2_G Number of Distance or Angle Restraints on AtSite          9 Note
PLAT063_ALERT_4_G Crystal Size Likely too Large for Beam Size ....       0.99 mm
PLAT154_ALERT_1_G The s.u.'s on the Cell Angles are Equal ..(Note)      0.002 Degree
PLAT171_ALERT_4_G The CIF-Embedded .res File Contains EADP Records          6 Report
PLAT172_ALERT_4_G The CIF-Embedded .res File Contains DFIX Records         10 Report
PLAT174_ALERT_4_G The CIF-Embedded .res File Contains FLAT Records          1 Report
PLAT230_ALERT_2_G Hirshfeld Test Diff for    Cl2      --C16       .       5.1 s.u.
PLAT230_ALERT_2_G Hirshfeld Test Diff for    S1       --C1        .      14.0 s.u.
PLAT230_ALERT_2_G Hirshfeld Test Diff for    S1A      --C2        .       7.6 s.u.
PLAT230_ALERT_2_G Hirshfeld Test Diff for    S1A      --C4        .      10.7 s.u.
PLAT230_ALERT_2_G Hirshfeld Test Diff for    C1       --C3A       .      13.0 s.u.
PLAT230_ALERT_2_G Hirshfeld Test Diff for    C2       --C3        .       6.0 s.u.
PLAT301_ALERT_3_G Main Residue  Disorder ..............(Resd  1  )        38% Note
PLAT802_ALERT_4_G CIF Input Record(s) with more than 80 Characters          1 Info
PLAT811_ALERT_5_G No ADDSYM Analysis: Too Many Excluded Atoms ....          ! Info
PLAT860_ALERT_3_G Number of Least-Squares Restraints .............         14 Note
PLAT912_ALERT_4_G Missing # of FCF Reflections Above STh/L=  0.600         23 Note
PLAT933_ALERT_2_G Number of OMIT Records in Embedded .res File ...          6 Note
PLAT978_ALERT_2_G Number C-C Bonds with Positive Residual Density.         12 Info
```

```
   0 ALERT level A = Most likely a serious problem - resolve or explain
   0 ALERT level B = A potentially serious problem, consider carefully
   2 ALERT level C = Check. Ensure it is not caused by an omission or oversight
  19 ALERT level G = General information/check it is not something unexpected

   1 ALERT type 1 CIF construction/syntax error, inconsistent or missing data
   9 ALERT type 2 Indicator that the structure model may be wrong or deficient
   4 ALERT type 3 Indicator that the structure quality may be low
   6 ALERT type 4 Improvement, methodology, query or suggestion
   1 ALERT type 5 Informative message, check
```

Publication of your CIF

You should attempt to resolve as many as possible of the alerts in all categories. Often the minor alerts point to easily fixed oversights, errors and omissions in your CIF or refinement strategy, so attention to these fine details can be worthwhile. In order to resolve some of the more serious problems it may be necessary to carry out additional measurements or structure refinements. However, the nature of your study may justify the reported deviations from journal submission requirements and the more serious of these should be commented upon in the discussion or experimental section of a paper or in the "special_details" fields of the CIF. *checkCIF* was carefully designed to identify outliers and unusual parameters, but every test has its limitations and alerts that are not important in a particular case may appear. Conversely, the absence of alerts does not guarantee there are no aspects of the results needing attention. It is up to the individual to critically assess their own results and, if necessary, seek expert advice.

If you wish to submit your CIF for publication in Acta Crystallographica Section C or E, you should upload your CIF via the web. If you wish to submit your CIF for publication in IUCrData you should upload your CIF via the web. If your CIF is to form part of a submission to another IUCr journal, you will be asked, either during electronic submission or by the Co-editor handling your paper, to upload your CIF via our web site.

PLATON version of 30/01/2018; check.def file version of 30/01/2018

Datablock I - ellipsoid plot

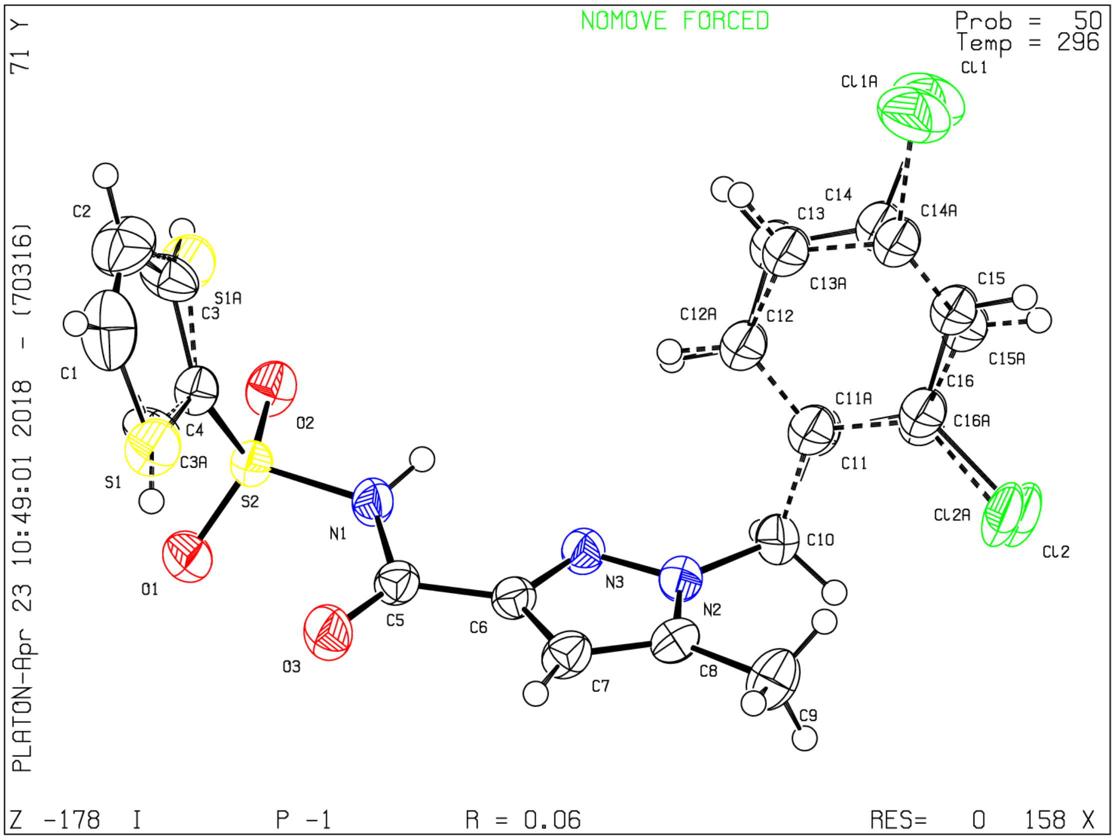

Supplement: Supplementary file 4 [file e-74-00747-sup4.pdf]
